# A Reaction-Diffusion Model to Capture Disparity Selectivity in Primary Visual Cortex

**DOI:** 10.1371/journal.pone.0024997

**Published:** 2011-10-13

**Authors:** Mohammed Sultan Mohiuddin Siddiqui, Basabi Bhaumik

**Affiliations:** Electrical Engineering Department, Indian Institute of Technology Delhi, New Delhi, India; University of Southern California, United States of America

## Abstract

Decades of experimental studies are available on disparity selective cells in visual cortex of macaque and cat. Recently, local disparity map for iso-orientation sites for near-vertical edge preference is reported in area 18 of cat visual cortex. No experiment is yet reported on complete disparity map in V1. Disparity map for layer IV in V1 can provide insight into how disparity selective complex cell receptive field is organized from simple cell subunits. Though substantial amounts of experimental data on disparity selective cells is available, no model on receptive field development of such cells or disparity map development exists in literature. We model disparity selectivity in layer IV of cat V1 using a reaction-diffusion two-eye paradigm. In this model, the wiring between LGN and cortical layer IV is determined by resource an LGN cell has for supporting connections to cortical cells and competition for target space in layer IV. While competing for target space, the same type of LGN cells, irrespective of whether it belongs to left-eye-specific or right-eye-specific LGN layer, cooperate with each other while trying to push off the other type. Our model captures realistic 2D disparity selective simple cell receptive fields, their response properties and disparity map along with orientation and ocular dominance maps. There is lack of correlation between ocular dominance and disparity selectivity at the cell population level. At the map level, disparity selectivity topography is not random but weakly clustered for similar preferred disparities. This is similar to the experimental result reported for macaque. The details of weakly clustered disparity selectivity map in V1 indicate two types of complex cell receptive field organization.

## Introduction

Humans and mammals with frontally located eyes see this world from different vantage points and the images formed on the left and right retinae differ. The difference in left and right retinal images is termed as binocular disparity. Binocular disparity can arise due to (i) difference in position between left and right retinal images and is encoded by receptive field (RF) positional disparity and phase disparity [1], (ii) difference in orientation between left and right retinal images called orientation disparity [2]–[4] and (iii) difference in spatial frequency in left and right retinal images called dif-frequency disparity [5], [6]. The visual system exploits binocular disparity to reconstruct 3D depth perception in vision. The neural mechanism specific to depth perception begins in V1, where processing of binocular signals first take place in cortical neurons. These cortical neurons encode binocular disparity of input stimuli for a small area of visual space [7]–[17]. Disparity selective cortical cells modulate their firing activity in response to binocular disparity of the stimulus in visual space.

In this paper we focus on disparity selective cortical cells that are orientation selective. If the left and right eye preferred orientations (ORs) differ, then this neuronal property is referred as interocular difference in preferred OR (IDPO) [2]. Blakemore et al. [4] have reported a range of ±15° (S = 6–9°) IDPOs in cat. Bridge & Cumming [2] have reported a range of ±20° (S = 9.22°) IDPOs in macaque. Cortical neurons encode orientation disparity through IDPOs to view 3D surface slants/tilts in visual space [4]. Also left and right eye preferred spatial frequencies (SFs) [18], [19] might differ. Psychophysical experiments report that difference in SF of left and right eye results in perception of slant-in-depth [20]–[22]. Binocular disparity caused by interocular SF difference is termed as dif-frequency disparity [5], [6]. In Stereopsis, the role of dif-frequency disparity is to perceive surface slants in depth.

For cortical cells with matched OR and SF in left and right eye but with horizontal and vertical offsets in their left and right Receptive field (RF) centers [7], [23] results in RF positional and phase disparities. Such cells encode disparity for vertical surfaces in visual space. RF positional disparity is the difference in center positions in left and right RFs having same subregion structures [16]. RF phase disparity occurs due to difference in subregion structures in left and right eye RFs but having same center positions [16]. Most often both RF position and phase disparities [1] are present.

In literature disparity selective cortical cells are analyzed at single cell level and then cell population data is studied. In adult cats, Ohzawa et al. [13] used drifting sine grating as left and right visual stimuli. The orientation and spatial frequency of sine gratings were kept at optimal values determined from left and right monocular tests. Cells with matching left eye and right eye orientation and spatial frequency preference were chosen for the study. The response of cortical cell as a function of interocular spatial phase difference between left and right sine grating stimuli were fitted with sine functions to determine its binocular interaction index (BII) or disparity sensitivity or disparity selectivity (DSen) [24]. BII (DSen) measures the degree to which the firing rate is modulated with respect to interocular spatial phase disparity of the inputs to left and right eye. BII (DSen) ≥0.3 indicates that the cells are spatial phase disparity selective. The interocular spatial phase disparity at which cortical cell response peaks determines the binocular preferred phase disparity. Later Freeman & Ohzawa [25] had reported cortical cellsâ€™ spatial phase disparity selectivity in kittens. Employing the experimental procedure by Ohzawa et al. [13], Chino et al. [26] had studied binocular interaction in catâ€™s cortical neurons and reported that at cell population level in cat (i) there is no correlation between preferred OR and BII (DSen) and (ii) there is no correlation between OD and BII (DSen) i.e. disparity selectivity. Recently, Kara & Boyd [24] had studied disparity selectivity in cat area 18 using drifting sine grating in left and right eye with varying interocular spatial phase disparity [13] and found no correlation between OD and disparity selectivity.

In cat, Ohzawa et al. [16] and later Anzai et al. [1] had studied RF position and phase disparities for cortical simple cells which modulate their firing rate with interocular spatial phase disparity. It was found that at cell population level (i) there is OR anisotropy of phase disparity: Wide range of RF phase disparity for cells tuned to near-vertical OR preference and narrow range of RF phase disparity for cells tuned to near-horizontal OR preference [1], [16] and (ii) slight positive correlation between RF position and phase disparities [1].

In macaque, Chino et al. [27] had studied disparity selectivity in V1 employing dichoptic sine grating in left and right retina with varying interocular spatial phase disparity [13] and found that preferred OR and BII (DSen) are independent of each other. Prince et al. [28] and Read & Cumming [29] employed random dot stereogram (RDS) as left and right visual stimuli with varying disparity between the two stimuli to study V1 cortical cells. Then they have computed BII and disparity discrimination index (DDI) as a measure of degree by which the firing modulation occurs with respect to disparity. They found no correlation between OD and DDI i.e. disparity selectivity.

In Cat V1, Anzai et al. [1] fitted 1D Gabor function to 1D RF profiles of left and right eye to assay the RF positional disparity and RF phase disparity for cortical cells. They found lower correlation between RF positional and phase disparities. In macaque V1, Prince et al. [30] fitted 1D Gabor function to disparity tuning curve to determine the RF position and phase disparity for cortical cells. They found slight positive correlation between RF positional and phase disparities. Tsao and Conway [31] reported an insignificant negative correlation between RF position and phase disparities in macaque by fitting 1D Gabor function to 1D RF profiles of left and right eye.

In Macaque V1, Prince et al. [28] have found that preferred disparities of multi- and single-unit recording from same location are weakly correlated. This shows disparity selectivity topography in V1 is not random but weakly clustered for similar preferred disparities in V1. V2 in monkey possesses a more highly organized representation for binocular disparity [32]. In cat area 17 so far no report on binocular disparity organization is reported. Kara & Boyd [34] have reported micro-architecture of disparity map in vertical OR preference sites in area 18 of cat visual cortex.

Though substantial amount of experimental data on disparity selective cells in V1 is available, no model on RF development for such cells or organization of disparity selective cells in V1 is available in literature. To the best of our knowledge, only one previous model deals with development of disparity selectivity in V1. Berns et al. [33] correlation model develops 1D RF of cortical cells with combined OD and disparity selectivity features using prenatal and postnatal development phases. Their results show zero disparity for binocular cells and non-zero disparity for monocular cells. Experimental studies of cat and macaque show no such relationship between OD and disparity selectivity at cell population level in V1 [26], [28], [29]. Other existing binocular receptive field (RF) models develop OR selectivity and OD features with/without directional selectivity and the corresponding maps across the cortex [34]–[42]. But they have not address disparity selectivity in their models.

Biological findings by Chino et al. [27] suggest that prenatal processes mostly determine disparity selectivity in cortical neurons. In this article, we present a pre-eye opening reaction-diffusion two-eye model to develop disparity selectivity in layer IV of cat V1. From our model we obtain left and right eye specific RFs for disparity selective simple cells. We obtain the spike response of these cells using a visual pathway model consisting of retina, LGN and cortical layer IV. In our modeled cortex 48.6% cells show disparity selectivity for vertical surfaces, 49.5% cells show dif-frequency selectivity i.e theses cells encode depth for slanted surface and 30.7% cells show significant IDPOs.

In this paper we focus on characterizing the model cells that are disparity selective for vertical surfaces. Our modeled cells that are disparity selective for vertical surfaces capture the following experimentally observed results.

1. Matched OR preference with interocular OR difference <±18° in both eyes [4].
2. Matched SF preference with interocular SF difference ≤±0.05 cycles/deg. in both eyes [18], [19].
3. Range of OD from left eye preference to binocular to right eye preference.
4. Lack of correlation between disparity selectivity and OD at cell population level as observed experimentally [26], [28], [29].
5. OR anisotropy of RF phase disparity [1], [16].
6. Slight positive correlation between RF position and phase disparities [1], [30].

At cortical map level, we have jointly developed OR, OD and disparity maps. OD peak points are located on/near the pinwheel singularities of OR map as observed experimentally by Crair et al. [43]. Disparity selectivity topography in our model V1 is not random but weakly clustered for similar preferred disparities. The map consists of disparity selective simple cells. The details of weakly clustered disparity selectivity map can provide insight into how disparity selective complex cell receptive field is organized from simple cell sub units. Receptive field structure of orientation selective complex cells is well studied. However, except some recent work [44], [45] not much is known regarding how simple cell sub-units are spatially pooled to form receptive field of a disparity selective complex cell. Our simulated map can be used to study possible receptive field organization of complex cells in V1. In absence of any experimental results on organization of disparity map in V1, our simulated map provides a window to study complex cell receptive field organization.

## Methods

### Three layer visual pathway model

**Figure 1:**
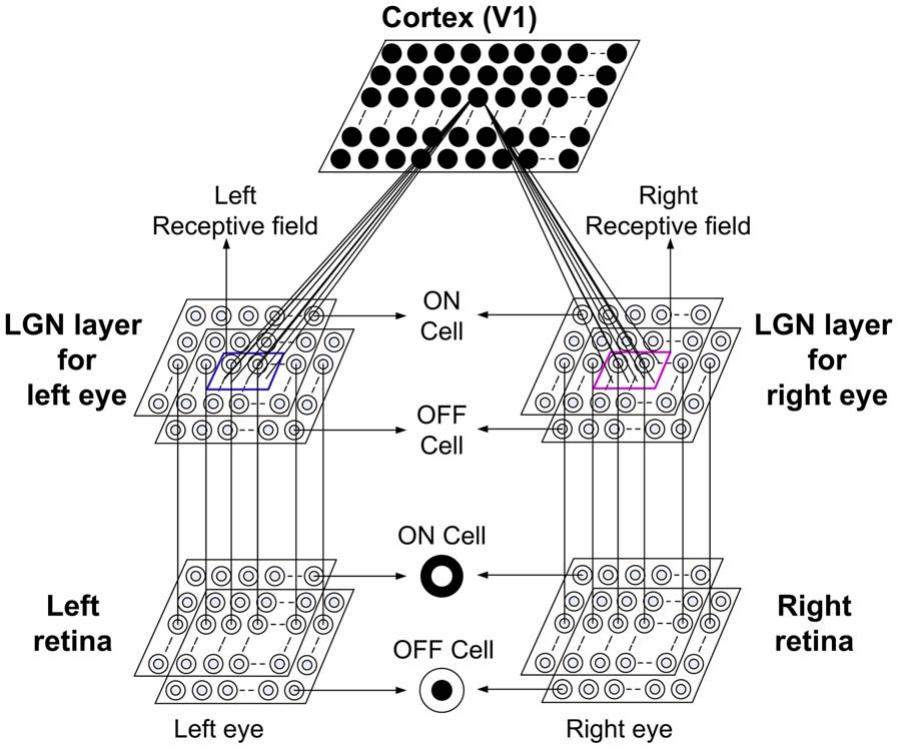
Three layer visual pathway model. (i) Layer 1: left and right retina/eye (each M x M overlapping ON and OFF retinal cells), (ii) Layer 2: left and right eye specific LGN layers (each M x M overlapping ON and OFF LGN cells), and (iii) Layer 3: IV layer of V1 in cat (N x N cortical cells). Each cortical cell in the model receives thalamic projections from each 13×13 left and right eye specific LGN cells centered at their retinotopic center. These thalamocortical connections define left and right RFs. We have used N = 50 and M = 30.

To obtain cortical cell response we have used a three-layer visual pathway model as depicted in Figure 1. The first layer models left and right retinae. Retina for each eye is modeled as two separate 2D 30×30 sheets of ganglion cells lying one over the other. One sheet corresponds to ON center and the other to OFF center ganglion cells respectively. Retinal ganglion cells (RGCs) have center-surround receptive field structure with center fields being 30′ wide [46] and center-to-center spacing between the cells being 12′ of the visual angle. The surround field was taken to be 90′ wide. The ganglion cell model used here has been used earlier [47]–[50] to produce realistic temporal responses to visual stimuli.

The second layer models left eye specific LGN layer and right eye specific LGN layer. Each LGN layer is also made up of two 2D 30×30 size sheets of LGN cells. One sheet comprised of ON center cells and the other of OFF center cells. It is reported that each LGN cell receives strong inputs from 1–3 retinal cells [51], [52]. In our model we have assumed that each LGN cell receive input from one retinal cell. The firing of the retinal cells is directly relayed to the LGN layer. The normalization constant in Wörgötter & Koch's [48] model was chosen such that the model LGN cells firing rate matched experimental values [53] for 50% contrast sinusoidal grating input to retina. The maximum firing rate for LGN cell is 40 spikes/sec.

The third layer models a 50×50 cortical layer IV of cat V1. Each cortical cell receives synaptic connections from 13×13 left and right eye specific ON/OFF LGN regions centered at its retinotopic position. The 13×13 left and right synaptic connections define left and right RFs of a cortical cell. Thalamic projection of 13×13 LGN cells corresponds to inputs from approximately 4° × 4° visual space. We have used a modified [50] SRM (Spike Response Model) [54] for obtaining cortical cell response. Details of the SRM model are given in File S1.

We have used our thalamo-cortical synaptic weight development model, presented in the next subsection, to obtain the connections between LGN and cortical cells. Biologically plausible competition and cooperation principles are used to model growth and decay of thalamo-cortical synaptic strengths. Both competition (reaction) and cooperation (diffusion) involves release of neurotrophic factors, neurotrophins which are activity-dependent [55]–[58]. We employ pre-eye opening environment with LGN spontaneous neural activity with characteristics as reported in Weliky & Katz [59] and assume cortical cells to be active during synaptic weight/strength update.

### Thalamo-cortical synaptic weight development: Model assumptions

The model is based on biologically plausible assumptions:

1. A pre-synaptic LGN cell gets connected to a number of cortical cells through pre-synaptic connections. Number of pre-synaptic connections a LGN cell supports is constrained by its pre-synaptic resource. A competition exists for a pre-synaptic resource where a pre-synaptic cell has a fixed amount of resource to distribute among its branches.

2. A post-synaptic cortical cell supports limited number of pre-synaptic connections depending on its post-synaptic resource. A competition exists between pre-synaptic LGN axons to get connected to post-synaptic cortical cell. The LGN axons compete for neurotrophic factors released by the post-synaptic cell.

Such fixed pre- and post-synaptic resources in retinal ganglion cell of gold fish [60], and optic tectum cell [61] are reported in literature.

**Figure 2:**
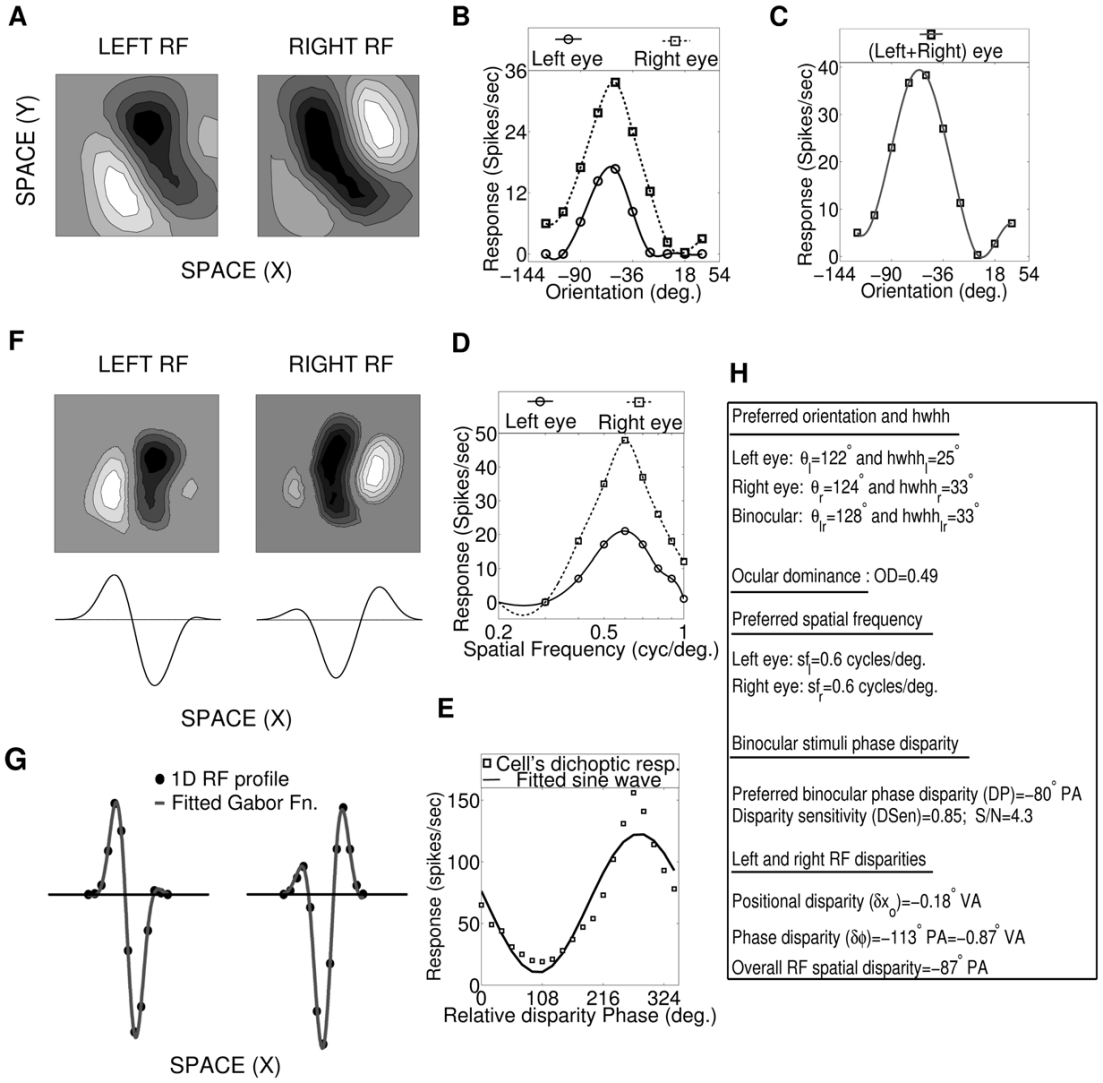
Simple cell response characterization. (A) Left and right 2D spatial (X-Y) RFs of a sample cortical cell from our 50×50 cortex. The ON and OFF subregions are shown in Grayscale with white (black) color representing strong synaptic connection from ON (OFF) LGN cells. The shading is proportional to the strength of the ON/OFF synaptic connections from LGN cells. (B) Left and right monocular OR tuning curves of the cell in ‘A’. Right eye response (maximum = 34 spikes/sec) dominates over left eye response (maximum = 17 spikes/sec). The OR preferences in left and right eyes are 122° and 124° (OR preference difference = 2°), with hwhh of 25° and 33° respectively. Eye preference i.e OD is 0.49. (C) Binocular OR tuning curve of the cell in ‘A’. The binocular OR preference is 120° with hwhh of 38°. (D) Left and right SF tuning curves of the cell in ‘A’. The optimal SF in left and right eye are 0.6 and 0.6 cycles/degree respectively. (E) Disparity tuning curve for the cell in ‘A’. The DP is −80° PA, DSen is 0.85 and S/N is 4.3. (F) 2D left and right spatial RFs of the cell in ‘A’ with X-axis transformation such that X-axis is orthogonal to cell's preferred orientation. 1D RF profiles is shown below the X-axis transformed 2D RFs. (G) 1D RF profiles marked with dark filled circles and fitted Gabor functions with solid curves for the cell in ‘A’. The positional disparity (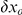) is −0.18° VA and phase disparity (*δφ*) is: −113° PA and −0.87 VA. The overall RF spatial disparity is −87° PA (H) Summary of the characterization of the sample cell in ‘A’.

3. Diffusive cooperation between near neighbors: (i) Post-synaptic cortical cells and (ii) same type of left (right) ON-ON and OFF-OFF pre-synaptic LGN cells. Experimental studies have shown that synaptic enhancement is not restricted to be specific to synapses where synchronous pre- and post-synaptic stimulation occur. But is also accompanied by spread of potentiation in two forms (see Figure 2 in Bi & Poo [62]): (i) spread of potentiation on different post-synaptic cells made by same pre-synaptic cell axons [63]–[65]. (ii) spread of potentiation from same post-synaptic cell to different pre-synaptic cell axons [66], [67]. These two forms suggest potentiation spread or cooperation between near neighbor post- and pre-synaptic cells influencing synaptic enhancement. This cooperation is modeled by diffusion terms in our model.

### Synaptic connection development from left and right specific LGN to cortex

In our model, 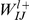 (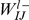) and 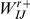 (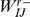), represents the strength of the connection from the ON (OFF) center LGN cell at position ‘J’ in left and right eye LGN layer respectively to the cortical cell at position ‘I’ in the cortical layer. Synaptic connection development from left eye specific LGN to cortex is governed by the equation given below:
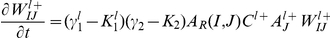


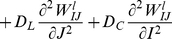
(1)where, 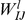 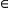
{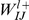, 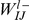}. In simple cells in layer IV from a given location in LGN there is a connection to a cortical simple cell either from an ON LGN cell or from an OFF LGN cell. 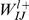 is represented as positive number and 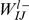 by negative number during simulation.

The term (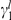
–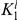) enforces competition for resources among axonal branches in a left eye specific LGN cell. 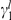 is the total presynaptic resource available in the left LGN cell at location ‘J’. 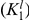 represents the presynaptic resources already consumed at location ‘J’. 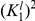 = 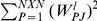 is the sum of square of synaptic strength of all branches emanating from the LGN cell at the location ‘J’. N x N is the size of cortex layer.

Similarly (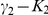) enforces competition among LGN cells for target space in the cortex. 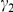 is the total postsynaptic resource available at cortical cell at location ‘I’. 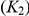 represents the postsynaptic resources already consumed at that ‘I’ location. 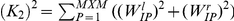 is the sum of square of synaptic strength of all branches of left and right eye LGN cells converging on the cortical cell at location ‘I’. M x M is the size of LGN layer. Heterosynaptic effects of stimulating an axon on other synaptic terminals innervating the same cell and resulting competition among synaptic terminals are well documented [68]–[70] in muscle cells. We have used N = 50 and M = 30.


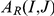
 is arbor function [71]. The arbor function defines the region from where a cortical cell receives its initial unorganized thalamic afferents. The amount of afferents a cell receives is determined by the arbor window. A trapezoidal window [71], where the window height reduces as one move towards the periphery of the window, has been used for the results reported here. A square window where the window height is unity inside the arbor and falls to zero at the arbor boundary can also be used. One of us has earlier [50] shown that RF structure does not depend on type of window used, be it trapezoidal or square. In case of square window, we obtained slightly better length to width ratio in receptive fields sub regions and it resulted in improved orientation tuning [50].

Left and right RFs of a cortical cell have subregions or subfields correspondence [16]. While updating 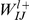, subregions correspondence is achieved by taking
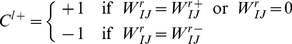
(2)


For *C^1+^* = +1, from LGN location ‘J’ synaptic connections from both left and right eyes are ON type. The presynaptic inputs from left and right eye specific LGN cells at ‘J’ add at the postsynaptic cell and 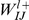 grow. For *C^1+^*  = −1, synaptic connection from left eye is ON type but synaptic connection from right eye is OFF type. Thus both the presynaptic inputs are not active at the same time and we assume that 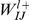 decays.

Our model employs pre-eye opening environment as LGN cell activity for synaptic weight development. 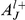 is the activity of ON center left eye specific LGN cell in location ‘J’. We have used the following LGN cell activities: (i) While updating a synaptic weight between a cortical cell and an LGN cell that particular LGN cell must be active. For instance while updating synaptic weight from the ON center LGN cell at position ‘J’ in left eye specific LGN, we put that LGN cell activity 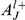 = 1. (ii) Activity of the LGN cell (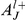) during synaptic weight update is determined by LGN spontaneous activity pattern as modeled by Goodhill [72]. If an LGN cell is inactive during weight update then the corresponding synaptic weight may decay unless helped by neighboring same-type cells.

Retrograde messengers are thought to be behind presynaptic spreading of synaptic strength enhancement for distances below 70 *µ*m [66]. Let us consider two neighboring synapses on two different dendritic branches. (i) Let the two dendritic branches belong to same postsynaptic cell (cortical cell) and the two synapses be formed by two neighboring presynaptic cell. If the two presynaptic cells are of same type i.e. both are ON LGN cells or both are OFF LGN cells, presynaptic spreading of synaptic strength will results in cooperation between two neighboring LGN cells. On the other hand if the two presynaptic LGN cells are of different type i.e. one is an ON LGN cell and the other is an OFF LGN cell, heterosynaptic inhibitory interaction will result. Heterosynaptic inhibitory interaction is suggested [73] as a potential mechanism for competition between co-innervating inputs. Molecular basis for correlation among ON- and OFF- center input to cortical cells are thought to be through NMDA receptor activation [74]. We assume here that the potentiation of a synapse (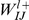) between an ON center LGN and a cortical cell is helped by presence of neighboring ON center LGN synapses but retarded by presence of OFF center LGN synapses. This is modeled through the second term on the RHS of equation (1). *D_L_* is the LGN diffusion constant. (ii) If the two dendritic branches belong to two neighboring postsynaptic cells (cortical cells) and the two synapses are formed by same presynaptic cell (an ON or an OFF center LGN cell), presynaptic spreading of strength would result in cooperative interaction between the two neighboring postsynaptic cells (cortical cells). This is modeled through the third term on the RHS of equation (1). *D_C_* is the cortical diffusion constant.

Cortical diffusion, *D_C_* ensures that near neighboring cells have similar RFs and OR preferences [75]. The number of sub fields in the RF of a cortical cell increases as *D_L_* is reduced. Effect of model parameters *D_C_ D_C_*, *D_L_*, LGN resource *γ*_1_ and cortical resource *γ*_2_Â on RF formation and response of cortical cells are similar as reported in Bhaumik & Mathur [50].

A similar differential equation is used for updating 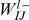. Similarly, synaptic connection development from right eye specific LGN to cortex 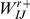
(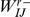) is modeled by replacing ‘l’ in weight updating differential equation (1) by ‘r’.

### RF development

In present study, we have modeled central visual field in layer IV in cat V1. In the peripheral visual field horizontal and vertical disparities ranges are similar [76]. However in the central visual field, there exist anisotropy between horizontal and vertical disparity [77]. Barlow et al. [7] found a 3∶1 ratio of horizontal to vertical position shift widths for cells between 5° and 15° eccentricities. Joshua and Bishop [23] found a 2.3∶1 ratio of horizontal to vertical position shift widths for cells between 8° and 12° eccentricities. Von der Heydt et al. [78] also found a bias toward larger horizontal than vertical position shifts for cells at 5°-10° eccentricities. In our model for development of receptive field, the relative distance between left and right RFs centers were randomly distributed with horizontal (H) shifts (-3≤H≤+3) and vertical (V) shifts (-1≤V≤+1) satisfying H∶V ratio of 3∶1 as reported in cat [7].

Each cortical neuron receives synaptic connections from left and right eye specific LGN layers. The initial synaptic weights are picked from uniform random distribution of weights of the order of 10^−6^. Synaptic connections are developed based on competition (reaction) and cooperation (diffusion) principle by employing our two-eye reaction-diffusion model equations with circular boundary condition. The number of presynaptic connections from a LGN cell to cortical cells depends on the presynaptic resource available with that LGN cell. We assume fixed presynaptic resource for each left (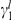) and right (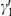) LGN cell in our model. The differential equations for updating synaptic connections are simulated in difference mode using synchronous weight update. Simulation was done with model parameters: *D_L_* = 0.0125, *D_C_* = 0.0075, 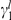 = 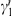 = 1 and *γ*_2_ = 1.5. The epochs were carried out till most of the resources, 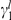, 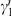 and *γ*_2_ are exhausted. At epoch 0, ON and OFF synaptic connections from left (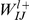, 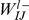) and right (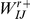, 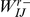) eye LGN cells, forming left and right RFs respectively are randomly organized. At around epoch 100, the left and right RFs of the cortical cells develop small patches, each patch being either ON or OFF synaptic connection from left and right LGN respectively. The formation of patches is due to cooperation effect among ON (OFF) synapses helping other neighboring ON (OFF) synapses to grow and push out any OFF (ON) synapses existing in the patch. This cooperation phenomenon is gradual and has come into existence due to diffusion in the LGN. At epoch 200, left and right RF structures of the cortical cells start to attain shape. At epoch 1000, left and right RF structures of the cortical cells have well defined segregated ON and OFF subregions. At epoch 3000, almost all available resources are consumed and developed RFs are well defined with gradual transition from ON/OFF subregion to other OFF/ON subregions (see Figure 2A).

## Results

### Response Characterization: Single cell

Now that the models for retinal cells, LGN cells, cortical cells, retina to LGN connections and LGN to cortical connections are in place, we simulated our model retina with sinusoidal grating and obtain cortical cell's spike response. The sinusoidal gratings are of 50% contrast at 0.5 cycles/degree spatial frequency and moving at a velocity of 2 degrees/second. The orientation of the input sinusoidal grating was varied from 0° to 180° in steps of 18°. The direction of motion of the grating was always orthogonal to the orientation of the grating. Each orientation was presented to the retina for thirty times. Peristimulus time histograms (PSTH) were made for each of the thirty presentations of an input stimulus. A bin width of 100 ms was used. Spike rates per second were computed for individual bins and the response was then averaged over the thirty-recorded Peristimulus time histograms. The cell spike response for any given orientation of input stimulus is the maximum response obtained in the averaged histogram. Ten responses were obtained for ten orientations of input stimulus. These ten responses are then converted into vectors having magnitude equal to response amplitude and angle equal to twice the angle of the grating. The OR preference of the cortical cell is half the angle of the resultant vector [79].

We characterize our developed cortical cells by ascertaining their OR selectivity, OD, SF preference, preferred binocular phase disparity, disparity selectivity (or sensitivity) and, left and right RF's offsets in terms of position and phase disparities. Figure 2A depicts left and right 2D spatial (X–Y) RFs of a sample cortical cell from our 50×50 cortex. The ON and OFF subregions are shown in gray-scale with white (black) color representing strong synaptic connection from ON (OFF) LGN cells. The shading is proportional to the strength of the ON/OFF synaptic connections from LGN cells. Figure 2B depicts left and right monocular OR tuning curves (see File S1) of the cell shown in Figure 2A. The right eye response (34 spikes/second) dominates over left eye response (17 spikes/second) in Figure 2B. The OR preferences in left and right eyes are 122° (with hwhh of 25°) and 124° (with hwhh of 33°) respectively. There exists a small OR preference difference of 2°. Eye preference i.e. OD is 0.49. Figure 2C depicts binocular OR tuning curve of the cell shown in Figure 2A. The binocular OR preference is 120° with hwhh of 38°. Figure 2D depicts SF tuning curves for left and right eye specific RFs of the cell shown in Figure 2A. The optimal SFs for left and right eyes are 0.6 and 0.6 cycles/degree respectively.

Figure 2E depicts response of a cortical cell as a function of relative phase difference between left and right eye dichoptic stimuli for the cell shown in Figure 2A. The phase disparity tuning shown in Figure 2E has smooth transition from suppression to facilitation. The cell response is maximally suppressed to 19 spikes/sec at a relative phase difference of 108° and maximally facilitated to 156 spikes/sec at a relative phase difference of 280° (−80°). Preferred binocular phase disparity is the relative phase difference between the left and right eye dichoptic sinusoidal grating stimuli at which a cortical cell fires most vigorously. The DP of the cell is −80° in phase angle (PA). Disparity selectivity or sensitivity (DSen) is calculated by fitting a sinusoidal curve to the cell response data. The ratio of the amplitude of the sinusoid used to fit the disparity tuning plot to its mean response amplitude is defined as disparity selectivity or sensitivity (DSen) [24] or Binocular interaction index (BII) [13]. The sinusoidal fitting is shown in Figure 2E. DSen for this cell is 0.85. The error in the fitting is expressed as ratio of amplitude of fitted sinusoid to the residual root mean square error of the fit and has been termed as S/N by Ohzawa and Freeman [13]. S/N of the cell is 4.3.

Developed simple cell RFs shown in Figure 2A have both position and phase disparities. These differences in the spatial structures of left and right RFs of the cell can be more apparently assayed through its 1D RF profiles [16]. 1D RF profiles are obtained by first transforming left and right 2D RFs of the cell such that the X-axis is orthogonal to cell's preferred OR. Then we integrate this transformed 2D RFs along their Y-axis to obtain 1D RF profiles. Figure 2F depicts 2D left and right RFs of the cell shown in Figure 2A with X-axis transformation such that X-axis is orthogonal to cell's preferred OR. In Figure 2F the RFs subregions of the cell are always elongated along the vertical axis, irrespective of their actual OR because of X-axis transformation. 1D RF profiles are shown below the X-axis transformed 2D RFs. To determine the difference in spatial structures of left and right RFs in terms of position and phase disparities, a 1D Gabor function is fitted to 1D profile of left and right RFs (see File S1). Figure 2G shows the 1D RF profiles marked with dark filled circles and fitted 1D Gabor functions with solid curves for the cell shown in Figure 2A. The positional disparity (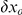) is −0.18° in visual angle (VA) and phase disparity (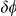) is −113° in PA and −0.87° in VA. Position and phase disparity together contributes to cell's overall RF spatial disparity. The overall RF spatial disparity is −87° PA. It is to be noted that overall RF spatial disparity (−87° PA) is closely equal to preferred binocular phase disparity (−80° PA). This reinforce that preferred binocular phase disparity of a cortical cell is a function of its RFs spatial offsets: Position and phase disparities. Figure 2H summarizes the characterization of the sample cell shown in Figure 2A.

**Figure 3:**
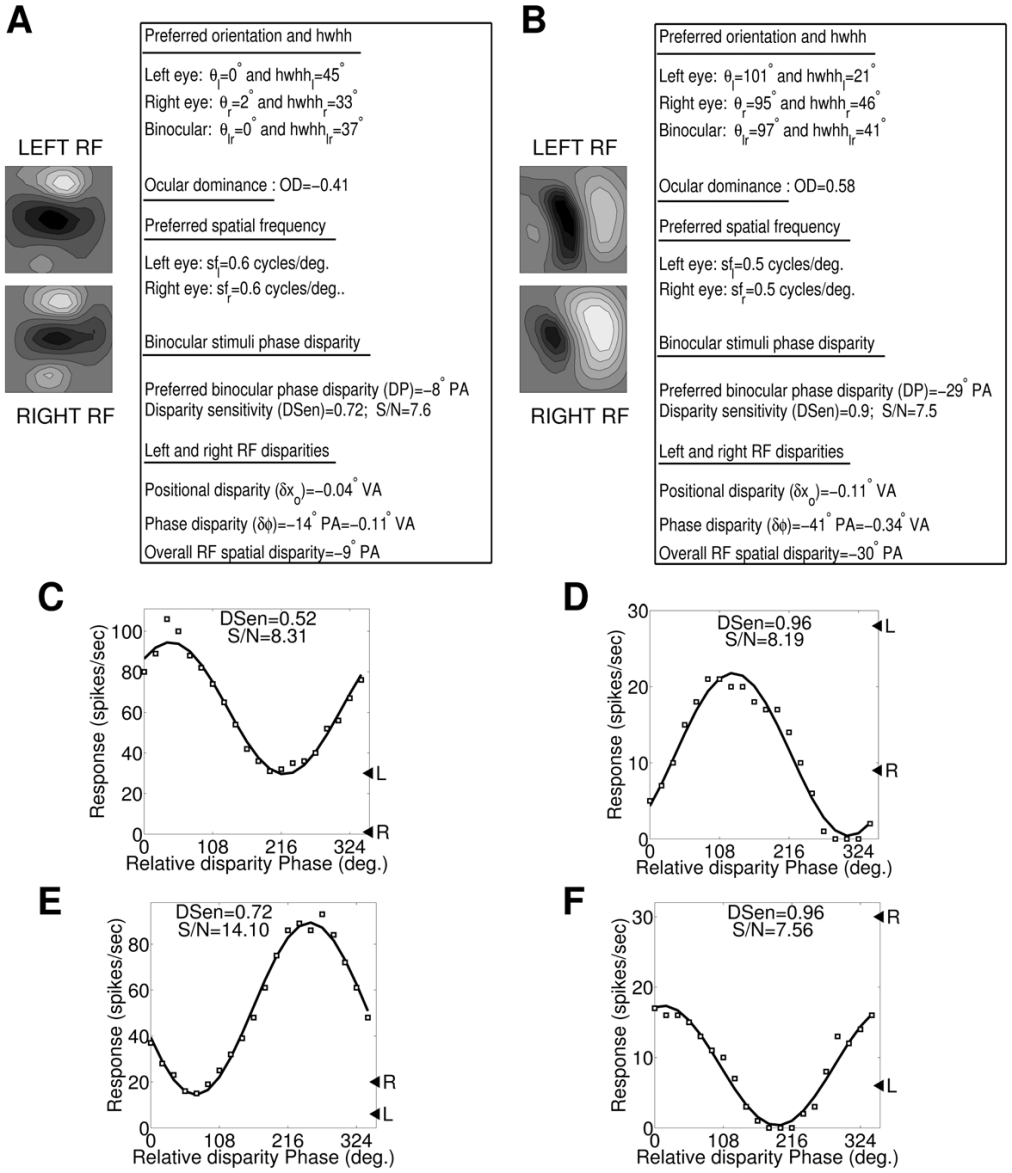
Binocular interaction in almost monocularly driven simple cells shows robust disparity selectivity. (A, B) Two sample cortical cells from our model cortex with left and right RFs along with their response characteristics and disparity values tabulated in table. (C, D) Phase disparity tuning curves for two sample cortical cells from our model. For both these cells, left monocular response (shown as ◂ L) dominates over the right monocular response (shown as ◂ R) with OD values of -1 and -0.83 respectively. The OD values closer to -1 categorize them as monocular left eye driven cells. These cells show robust binocular interaction with DSen (S/N) values of 0.52 (8.31) and 0.96 (8.19) respectively. The nature of binocular interaction is synergistic for the first cell (Figure 3C) and suppressive for the second cell (Figure 3D). (E, F) Phase disparity tuning curves for two sample monocular cortical cells with OD values close to +1. For both these cells, right monocular response (shown as ◂ R) dominates over the left monocular response (shown as ◂ L) with OD values of 0.92 and 0.84 respectively. These cells show robust binocular interaction with DSen (S/N) values of 0.72 (14.1) and 0.96 (7.56) respectively. The nature of binocular interaction is synergistic for the first cell (Figure 3E) and suppressive for the second cell (Figure 3F).

Figure 3(A, B) depicts two other sample cortical cells from our model cortex with left and right RFs. The response characteristics and disparity values of these two cells are shown in a table besides their RFs. In similar manner we characterize all cells in our model cortical layer IV. Disparity selective cortical simple cells are basically driven by both eyes i.e. they are binocular driven cells. Electrophysiological studies [26], [27] report that even almost monocularly driven simple cells shows binocular interaction with robust disparity sensitivity or selectivity (DSen≥0.3 and S/N>2 [13]). The nature of binocular interaction may be either synergistic or suppressive. In our model cortex too, almost monocularly driven simple cells show binocular interaction with robust disparity selectivity. Figures 3C and 3D depict phase disparity tuning curve for two sample cortical cell from our model cortex. For both these cells, left monocular response (shown as ◂ L) dominates over the right monocular response (shown as ◂ R) with OD values of -1 and -0.83 respectively. The OD values closer to -1 categorize them as monocular left eye driven cells. Nonetheless these cells show robust binocular interaction with DSen (S/N) values of 0.52 (8.31) and 0.96 (8.19) respectively, as depicted in Figures 3C and 3D. The nature of binocular interaction is synergistic for the first cell (Figure 3C) and suppressive for the second cell (Figure 3D). We also obtained similar binocular interaction with robust disparity selectivity for almost monocularly right eye driven cells. Monocularly right eye driven cells have OD values closer to +1. Figure 3(E, F) depicts binocular interaction having synergistic and suppressive nature for almost monocularly right eye driven cells (OD = 0.92 and OD = 0.84).

### Cell population response

**Figure 4:**
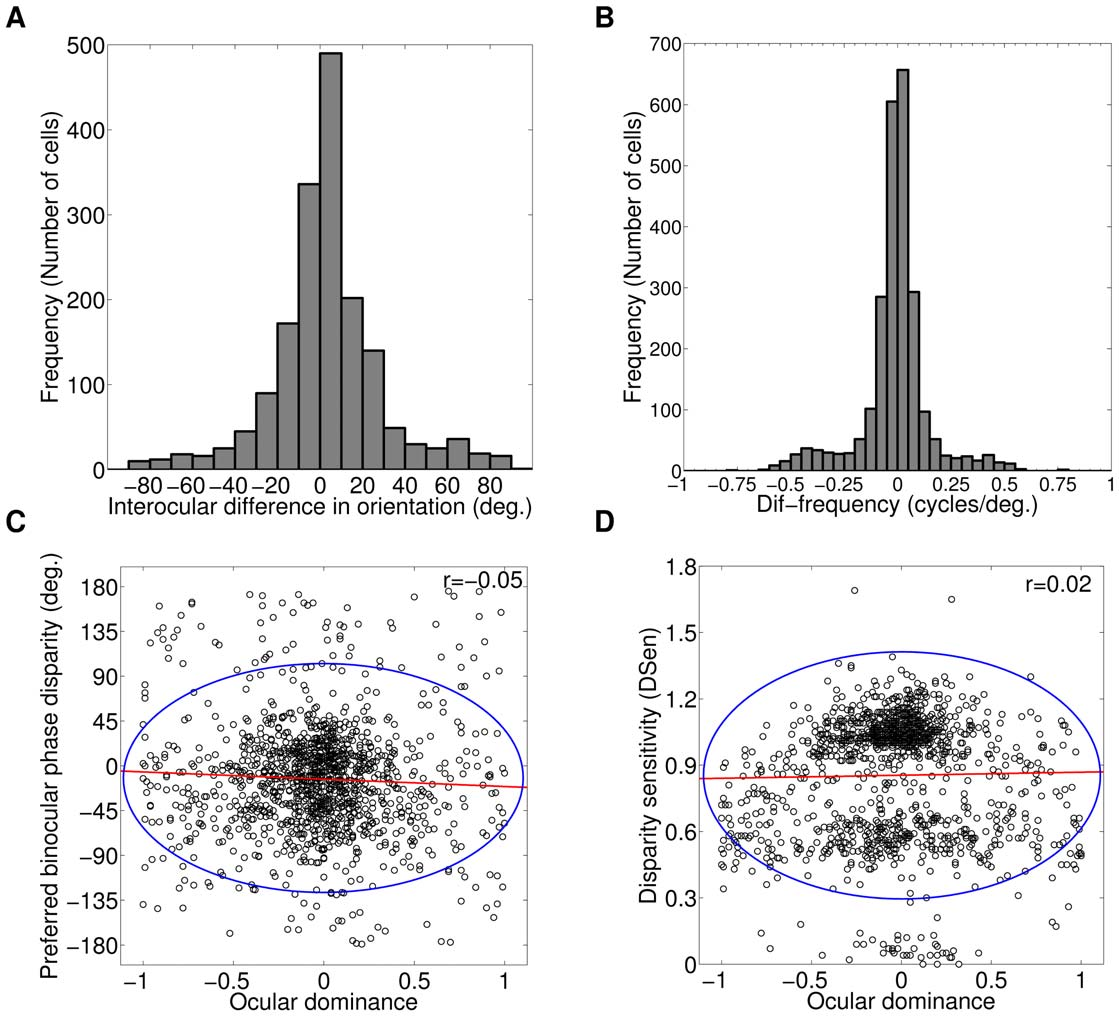
IDPO, Dif-frequency, OD versus DP, and OD versus DSen. (A) Histogram of interocular difference in preferred ORs. In our model cortex, 69.3% OR tuned cells (1200 out of total 1732) have IDPOs range of ±20° (S = 9°). Rest 30.7% of cells have significant IDPOs (>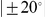). (B) Histogram of Dif-frequency cells in cycles/degree. In our model cortex, 50.44% of cortical cells (1261 out of total 2500) have Dif-frequency range of ±0.05 cycles/degree. In remaining 1239 cells, 1179 (47.16%) cells have significant Dif-frequency (>
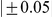
0.05 cycles/degree) and 60 (2.4%) cells are OR untuned in both eyes or have very weak responses from which we were not able to determined there SFs in left and right eyes. (C) Scatter plot of preferred binocular phase disparity (in −180° to +180° scale) and OD for all OR tuned cells. The plot shows almost no correlation (r = -0.05) between them at cell population level. (D) Scatter plot of disparity sensitivity (or selectivity) and OD for all OR tuned cells. The plot shows almost no correlation (r = 0.02) between them at cell population level. The red line represents the linear regression line and blue ellipse indicates 95% prediction interval in ‘B’ and ‘C’.

In our 50×50 cortex, total number of OR tuned cells is equal to 1732 out of total 2500 cells accounting to 69.3% of OR tuned cells. Rest 30.7% cells are OR untuned in at least one eye. OR tuned simple cells may possess difference in their left and right eye preferred ORs. This neuronal property is referred as interocular difference in preferred ORs (IDPOs) [2]. Blakemore et al. [4] have reported a range of ±15° (standard deviation (S) = 6–9°) IDPOs in cat. Bridge & Cumming [2] have reported a range of ±20° (S = 9.22°) IDPOs in macaque. In our model cortex, 69.3% OR tuned cells (1200 out of total 1732) have IDPOs range of ±20° (S = 9°). Rest of the cells has significant IDPOs (>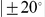). Figure 4A depicts histogram of IDPOs in degrees. In our model cortex, we ascertain SF for cortical cells which are atleast OR tuned in one eye. Our simulated cells with model parameter *D_L_* = 0.0125 have SF range of 0.2–0.85 cycles/degree. We can achieve a wider SF range of 0.19–1.04 cycles/degree by varying *D_L_* parameter in our simulation [80]. Experimental finding in cat reports SF range of 0.3–1.8 cycles per degree [81]. Our simulated cortical cells SF range lacks in covering high spatial frequencies as compared to experimentally observed SF range in cat. We attribute this difference to fixed centre size (30′) retinal X-cell employed in our model. In cat, retinal X-cell centre sizes varies from 20′ in the central area to about 40′ at an eccentricity of 0.75 mm (see Figure 7 in Peichl & Wässle [46]). Broader range of SF can be achieved by incorporating retinal X-cells with different centre sizes in our model.

Electrophysiological studies in cat [6], [16], [18] and monkey [19] have reported a population of cells with difference in SF of left and right eye. In our model cortex, 50.44% of cortical cells (1261 out of total 2500) have almost same spatial frequency in both eyes (Dif-frequency range of ≤±0.05 cycles/degree). In remaining 1239 cells, 1179 cells have significant Dif-frequency (>
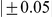
cycles/degree) and 60 cells are OR untuned in both eyes or have very weak responses from which we were not able to determined there SFs in left and right eyes. Figure 4B depicts histogram of Dif-frequency cells in cycles/degree. Cortical cells with significant Dif-frequency (1179 out of total 2500) in our model can be used to detect surface slants [6].

In order to determine the phase disparity tuning characteristics of a cortical cell, the relative phase difference between left and right dichoptic stimuli is varied with same spatial frequency in both eyes. This means that cortical cells (1261 out of total 2500) that have well matched SFs in both eyes are the ones, which may possess phase disparity tuning characteristics. Such cells acts as fronto-parallel or vertical surface disparity detectors and referred generally as disparity selective cells if DSen≥0.3 and S/N>2 [13].

In this article we will concentrate only on disparity selective cells i.e vertical surface disparity detection cells. In our model cortex, 1215 out of 1261 cortical cells having same SF in left and right eye are disparity selective with S/N>2 and DSen≥0.3 (*µ* = 0.883, S = 0.243) (see File S1). Freeman & Ohzawa [25] have reported that for 3 and 4 week postnatal kittens and adults, the majority of simple cells shows phase specificity. Chino et al. [27] have also reported >70% disparity selective cells in 1-week old monkey.

Now we ascertain whether preferred binocular phase disparity and OD show any dependency at single cell level in our model cortex. This is found by estimating correlation between preferred binocular phase disparity and OD at cell population level. Figure 4C depicts a scatter plot of preferred binocular phase disparity (in −108° to +180° scale) and OD. The plot shows almost no correlation (r = −0.05) between them at cell population level. The red line represents the linear regression line and blue ellipse indicates 95% prediction interval.

Having known that preferred binocular phase disparity is not related to OD at cell population level, we check whether disparity sensitivity (or selectivity) show any bias with OD. To substantiate this, we obtain correlation between disparity sensitivity and OD. Figure 4D depicts a scatter plot of disparity sensitivity and OD for all OR tuned cells for which phase disparity tuning can be determined (1261 out of total 2500). The plot shows almost no correlation (r = 0.02) between them at cell population level. Several important aspects of disparity sensitivity (DSen) should be noted (1) DSen was generally stronger for relatively balance OD (OD range −0.33 to +0.33). The mean DSen value is 0.94. (2) Monocular cortical simple cells (0.67<
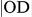
<1) or completely monocularly driven cortical simple cells (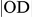 = 1) exhibit substantial binocular interaction with mean DSen value of 0.69. These results agree well with experimental finding by Chino et al. [26].

Cortical cells with near-vertical OR preference are well suited for detecting the horizontal disparity cues for 3D depth perception in vision [82]. To check whether a particular OR preference of a cortical cell has more bias towards disparity sensitivity, we estimate correlation between DSen and preferred OR for our model cortical cells. We found no correlation between cortical cell's DSen and preferred OR preference (r = 0.004). From this result, it is evident that cortical simple cells do not treat near-vertical OR preference differently for detecting horizontal disparity sensitivity. We also look into the correlation between OR bandwidth and DSen. We found no correlation between DSen and OR bandwidth for our model cortical cells (r = 0.085). These results conform to the experimental findings [13], [83].

**Figure 5:**
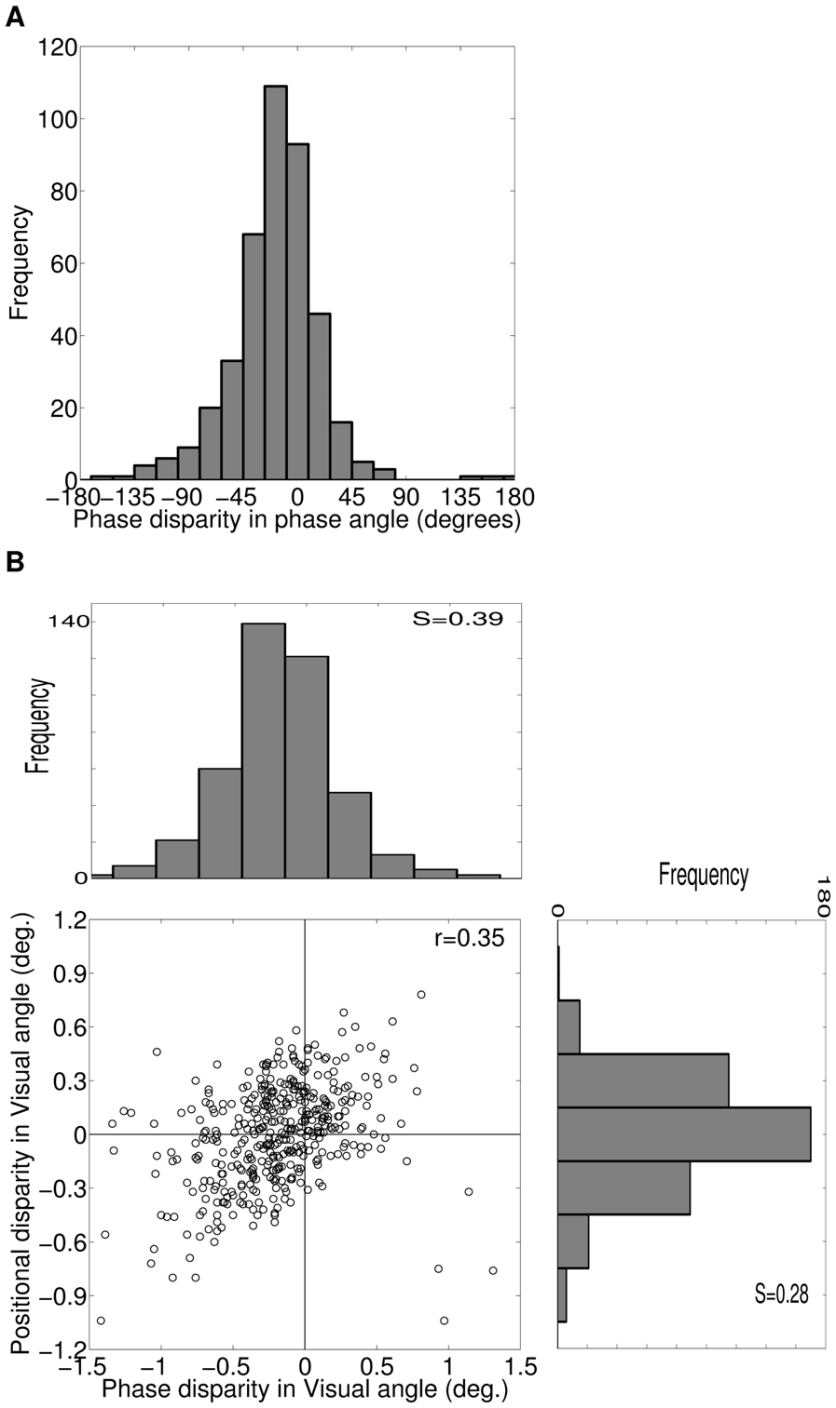
RF phase and positional disparities for 415 cells. (A) Histogram of RF phase disparity in PA. 25% of cells have phase disparities in the range −10° to 10° PA. Rest 75° cells have broader range of phase disparities. Overall 95.7% of cells has phase disparity in the range −90° to 90° PA. The phase disparities lies in the range of −162° to 180° PA. (B) Scatter plot of RF position disparity versus phase disparity in VA and their respective histograms. The RF position and phase disparities show slight positive correlation between them (r = 0.35). The total range of phase disparities lie within ±1.4° VA (S = 0.39). Total range of position disparities lie within ±1° VA (S = 0.28).

Out of total 1215 disparity selective cells in our model cortex we have obtained RF positional disparity, RF phase disparity and overall RF spatial disparity for 415 cells by fitting 1D Gabor function (see File S1). Figure 5A depicts histogram of RF phase disparity in PA. RF phase disparities in our simulated cells lies in the range of −162° to 180° PA. Prince et al. [30] have used RF phase disparity (*δφ*) of cortical cells to map them to the class of disparity tuned cells defined by Poggio [84]–[86] as: (1) Tuned excitatory (TE) cells having −45°<*δφ*<45°, (2) Tuned inhibitory (TI) cells having: −180°<*δφ*<−135° or 135°<*δφ*<180°, (3) Near (NE) cells having 45°<*δφ*<135° and (4) Far (FA) cells having −135°<*δφ*<−45°. Out of total 415 cells for which RF phase disparities were determined in our model cortex, 332 (80%) are TE cells, 5 (1.2%) are TI cells, 8 (1.9%) are NE cells and 72 (16.9%) are FA cells. It is evident from Figure 5A that RF phase disparity distribution varies smoothly between −180° to 180°. This suggests that disparity tuned cells in our model cortex: TE, TI, NE and FA, does not form distinct classes. This result is in agreement with experimental findings [30], [31] in macaque.

Next we have studied correlation between RF position and phase disparities. To do so, we have obtained RF phase disparities in VA because RF position disparities are generally expressed in VA. Figure 5B depicts scatter plot of RF position disparity versus phase disparity in VA and their respective histograms. The RF position and phase disparities show slight positive correlation between them (r = 0.35). Prince et al. [30] reported a slight positive correlation (r = 0.24) between RF position and phase disparities in macaque. Tsao & Conway [31] reported an insignificant negative correlation (r = −0.22) between RF position and phase disparities in macaque. Anzai et al. [1] reported lower correlation (r = 0.12) between RF position and phase disparities in cat. Range of phase disparities lie within ±1.4° VA (S = 0.39). Range of position disparities lie within ±1° VA (S = 0.28). The obtained range of positional and phase disparities in VA correspond roughly to the binocular fusion range in cats [87].

**Figure 6:**
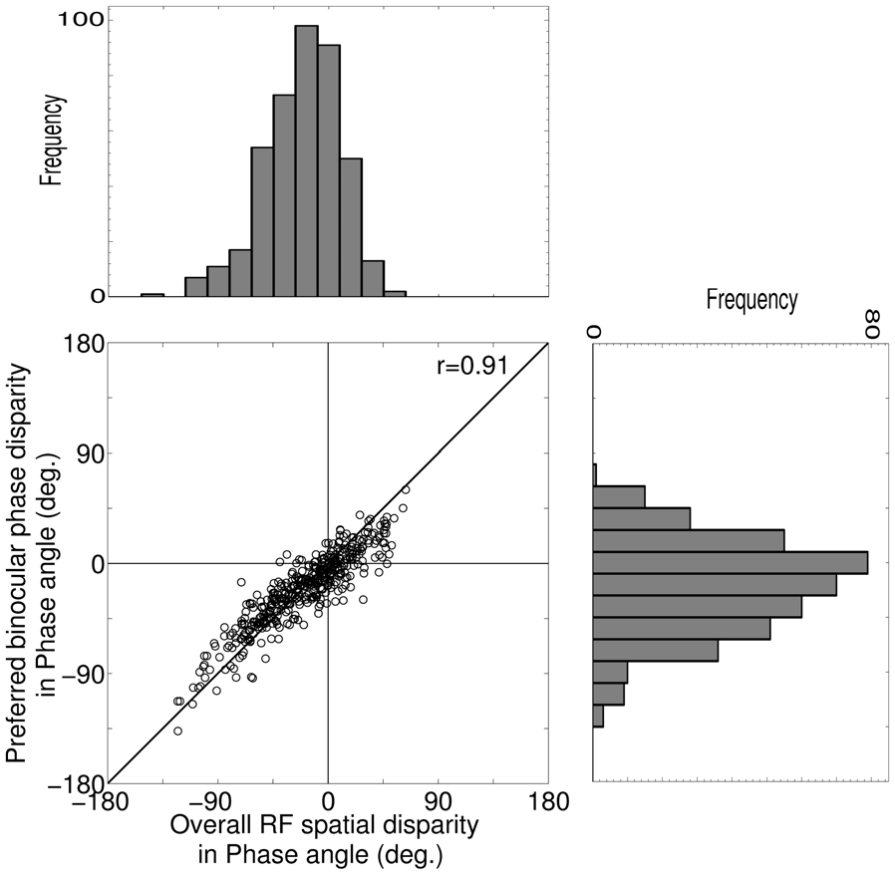
DP versus Overall RF spatial disparity for 415 cells. Scatter plot of preferred binocular phase disparity versus overall RF spatial disparity and their respective histograms. The preferred binocular phase disparity and overall RF spatial disparity shows strong correlation of r = 0.91.

The phase disparity tuning response of a disparity selective cortical cell as a function of relative phase difference between left and right dichoptic stimuli is mainly due to its overall RF spatial disparity between left and right eye RFs. This suggests that preferred binocular phase disparity and overall RF spatial disparity should be highly correlated to each other. To substantiate this notion, we ascertain the correlation between preferred binocular phase disparity and overall RF spatial disparity. Figure 6 depicts scatter plot of preferred binocular phase disparity versus overall RF spatial disparity and their respective histograms. As expected, preferred binocular phase disparity and overall RF spatial disparity shows strong correlation of r = 0.91.

### Maps

**Figure 7:**
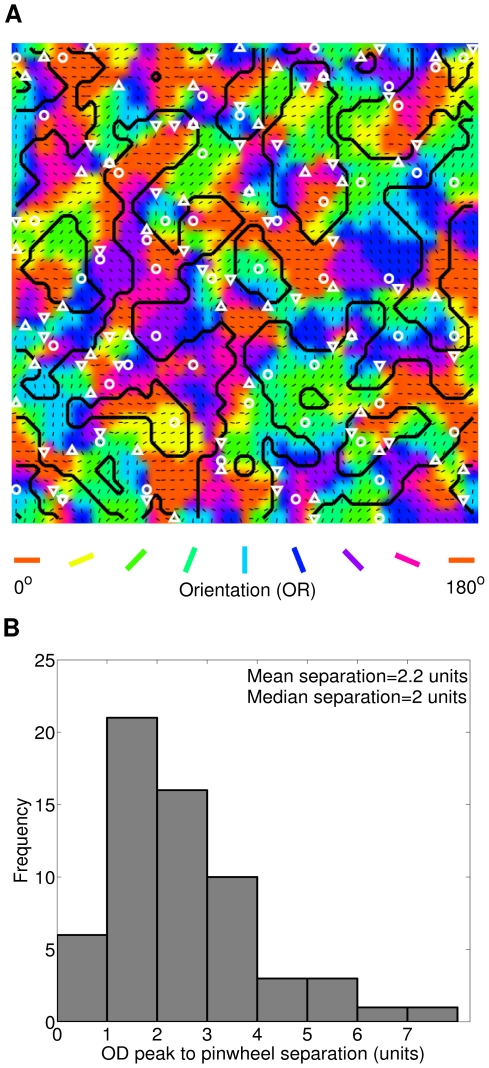
OR map superimposed with OD map contours. (A) Binocular OR map using color code scheme in 0° to 180° scale with superimposed OD map contours marked with thick black lines. The black color bars are oriented at cell's binocular preferred OR. The OD peak points are marked with white color circle markers. The pinwheel singularities are marked with: (i) white color up pointing triangle markers for positive pinwheel singularities and (ii) white color down pointing triangle markers for negative pinwheel singularities. The OD peak points appear on/near the pinwheel singularities. (B) Histogram of OD peak points to pinwheel separation across the model cortex. The mean separation is 2.2 units and median separation is 2 units.

Next we focus on OR, OD and disparity map in our model cortex. Figure 7A depicts binocular OR map having color code scheme in 0° to 180° scale with superimposed OD map contours marked with thick black lines. The black color bar markers are oriented at cell's binocular preferred OR. The OD peak points are marked with white color circle markers. The pinwheel singularities are marked with: (i) white colored up pointing triangle markers for positive pinwheel singularities and (ii) white colored down pointing triangle markers for negative pinwheel singularities. It is evident from Figure 7A that the OD peak points appear on/near the pinwheel singularities, conforming to the experimental finding by Crair et al. [43]. Figure 7B shows the histogram of OD peak points to pinwheel singularities separation. The mean separation is 2.2 units and median separation is 2 units.

**Figure 8:**
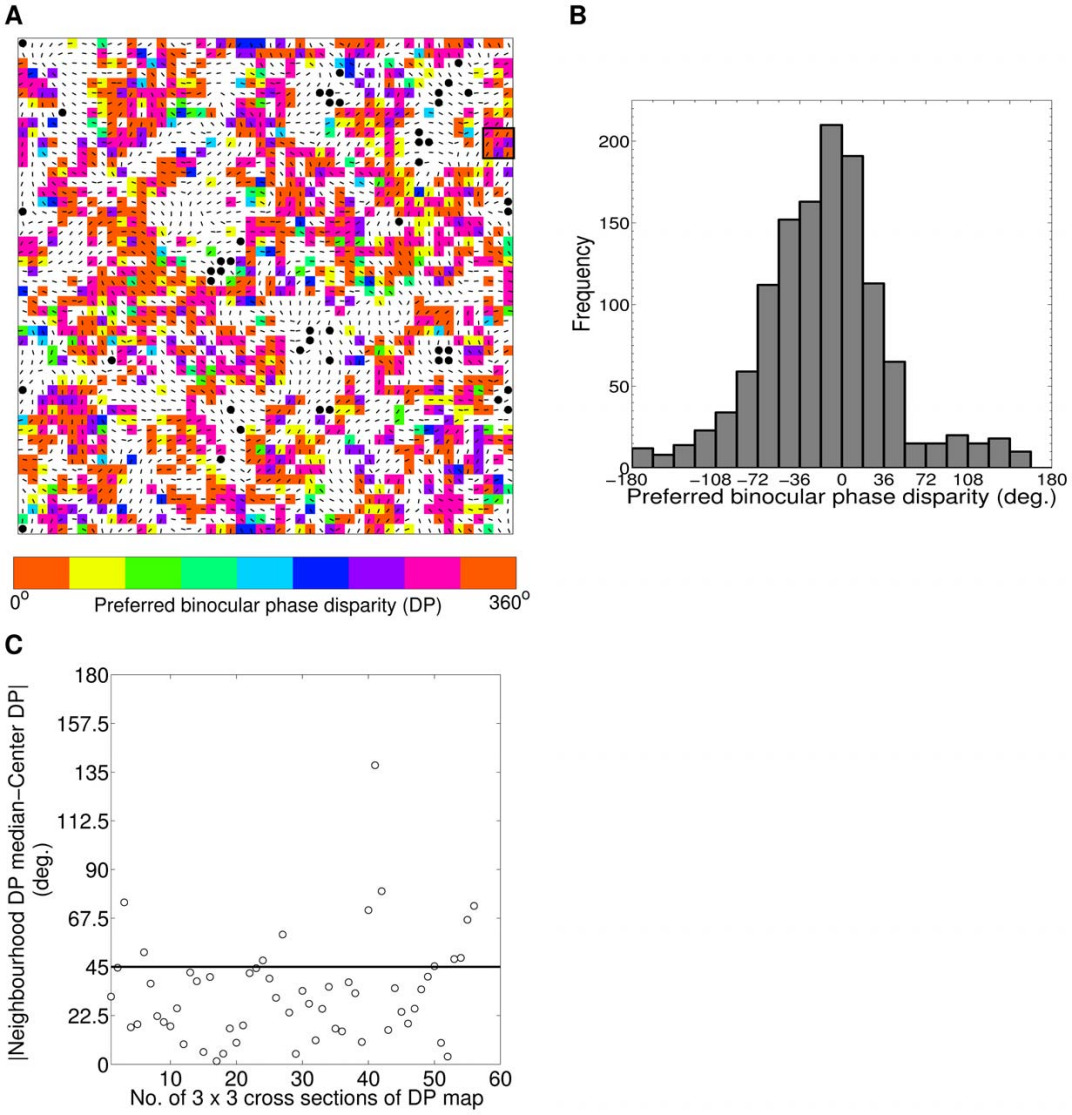
Disparity map and its organization. (A) Disparity map using color code scheme in 0 to 360 scale. The black color filled circles represents cortical cells with DSen¡0.3. The white color cells are Dif-frequency disparity selective cells. The superimposed oriented black color bars depict binocular preferred OR for cells in the map. (B) Histogram of DP across the model cortex in −180 to 180 scale. (C) Here we explored the smoothness of the topographic organization of DP by finding similarity/smoothness in 3×3 neighborhoods in DP map. A sample 3×3 section of DP map is shown in Figure 8A marked with black square boundary. Figure 8C depicts the difference between neighborhood median DP and center DP for 56 possible 3×3 sections from our DP map. In 45 out of total 56 possible 3×3 sections difference is ≤45° threshold difference. This result suggests that DP values in disparity map are weakly clustered together.

Disparity map captures the preferred binocular phase disparity (DP) of cortical cells (1215/1261) having DSen≥0.3 and S/N>2 across the cortex. Disparity map having color code scheme in 0° to 360° scale is shown in Figure 8A. The black color filled circles represents cortical cells with DSen<0.3. The white color cells are Dif-frequency disparity selective cells. The superimposed oriented black color bars depict binocular preferred OR for cells in the map. Figure 8B depicts histogram of preferred binocular phase disparity across the model cortex in −180° to 180° scale. 393 out of 1215 cells (32.3%) have phase disparities in the range −18° to 18° PA. Overall 86.5% (1051/1215) of cells has DP in the range −90° to 90° PA. Total DP lies in the range of −180° to 162° PA.

Prince et al. [28] reported that though disparity selectivity is not as highly organized as in OR map, topographic organization of disparity selectivity in V1 does not possess salt and pepper arrangement. Prince et al. [28] found a weak correlation between preferred disparities of multi- and single-unit recording in monkey. We have explored the smoothness of the topographic organization of DP in our disparity map by finding similarity/smoothness in 3×3 neighborhoods in DP map. We investigated 56 possible 3×3 sections in our DP map. A sample 3×3 section of DP map is shown in Figure 8A marked with black square boundary. A 3×3 section of DP map with center (x, y) is considered locally smooth/similar if the difference between median DP over the neighborhood of (x, y) and DP at (x, y) is below a threshold value of 45°. Figure 8C depicts the difference between neighborhood median DP and center DP for 56 possible 3×3 sections of our DP map. In 45 out of total 56 possible 3×3 sections difference is ≤45°. This result suggests that DP values in disparity map are weakly clustered together. Our result is in agreement with experimental finding by Prince et al. [28] in monkey.

### Complex cells

**Figure 9:**
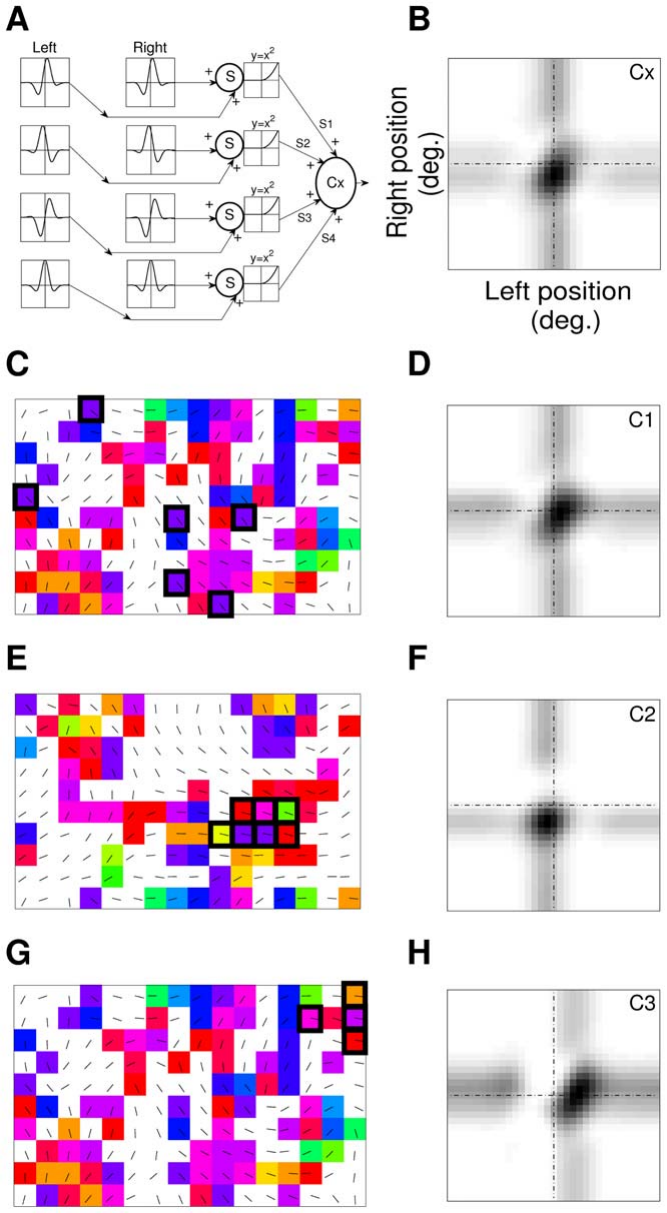
Complex cells. (A) Complex cell Cx built using four linear filters with spatial phases of −45°, 45°, −90° and 0°. (B) Binocular RF of complex cell Cx. (C) Complex cell C1 built using simple cell subunits with almost same disparity selectivity. The simple cell subunits are marked with black rectangular outlines in DP map patch. (D) Binocular RF of complex cell C1. (E) Complex cell C2 built using simple cell subunits exhibiting a systematic change in disparity selectivity. Subunits are marked with black rectangular outlines. (F) Binocular RF of complex cell C2. (G) Complex cell C3 built using simple cell subunits exhibiting a systematic change in disparity selectivity. Subunits are marked with black rectangular outlines. (H) Binocular RF of complex cell C3.

We now study the implications of the weakly clustered disparity selective simple cells on the receptive field formation of disparity selective complex cells. In the energy model for the receptive fields of complex cells [88], [89] two linear filters (simple cells) that are separated by 90° in spatial phase form a complex cell after filtering of visual stimuli and squaring operation. Later it was shown that four linear filters with squaring operation are needed [15], [90]. Instead of using standard four filters with spatial phase of 0°, 180°, −90° and 90°, when we use four linear filters with spatial phase of −45°, 45°, −90° and 0° (see Figure 9A) to construct a complex cell we obtain a receptive field as shown in Figure 9B. This minimalist schema is a fair approximation to most of the actual complex cells receptive field where pooling ratio ( =  RF size of complex cell/ RF size of simple cell subunit) is approximately 1.28 [45].

The receptive fields of complex cells in V1 are more circular and only slightly larger than their simple cell subunits in size [44]. As complex cell subunits occupy spatial extents similar to those of simple cell receptive fields we choose simple cell subunits that have same orientation preference and considerable RF overlap to build complex cells. From the spatial organization of the disparity selective simple cells in our model cortex we find two types of complex cell receptive fields. Complex cell RF consisting of simple cell subunits having (i) same/ almost same disparity selectivity and (ii) different disparity selectivity. Three complex cell RFs along with the cortical patch from where simple cell subunits are chosen are shown in Figure 9C to Figure 9H. The simple cells subunits for complex cell formation are marked with black rectangular outline in Figure 9C, Figure 9E and Figure 9G for three complex cells C1, C2 and C3 respectively. For each of the constituent simple cell subunits spatial phase was determined by fitting 1D Gabor function to 1D profile of left and right RFs of the cell (see File S1). Tables 1, 2, and 3 contain the details of the spatial phase and disparity values of simple cell subunits of three complex cells C1, C2 and C3. The RF of complex cell C1 shown in Figure 9D consists of simple cell subunits with almost same disparity selectivity and constituent subunits S1 & S4 and S3 & S6 have 90° phase difference as suggested in energy model. For Complex cell C1 we have used six subunits. Some studies point that more than four linear filters are required for constructing complex cell receptive fields [6], [91]. The complex cell C2 and C3 (see Figure 9F and Figure 9H) consist of simple cell subunits exhibiting a systematic change in disparity (see Table 2 and Table 3) unlike the same disparity for constituent subunits in energy model. Complex cells C2 and C3 can potentially signal inclination in the 3D space by the gradual shift of preferred disparity within the RFs. A detailed study of how at the V1 level complex cells pool activities of simple cells will be reported separately.

**Table 1:** Modeled simple cell 1D RF profile phases for complex cell C1.

Complex cell C1						
	S1	S2	S3	S4	S5	S6
**Left eye phase**	−0.29*π*	0.05*π*	−0.03*π*	−0.74*π*	−0.25*π*	0.48*π*
**Right eye phase**	−0.23*π*	0.38*π*	0.38*π*	−0.06*π*	−0.14*π*	0.8*π*
**DP**	288°	292°	296°	299°	303°	305°

**Table 2:** Modeled simple cell 1D RF profile phases for complex cell C2.

Complex cell C2							
	S1	S2	S3	S4	S5	S6	S7
**Left eye phase**	−0.17*π*	0.32*π*	0.28*π*	0.11*π*	0.31*π*	0.33*π*	0.15*π*
**Right eye phase**	0.4*π*	0.51*π*	0.54*π*	0.27*π*	0.66*π*	0.61*π*	0.39*π*
**DP**	295°	304°	336°	2°	9°	63°	96°

**Table 3:** Modeled simple cell 1D RF profile phases for complex cell C3.

Complex cell C3				
	S1	S2	S3	S4
**Left eye phase**	−0.75*π*	−0.37*π*	−0.92*π*	−0.73*π*
**Right eye phase**	−0.36*π*	0.37*π*	−0.69*π*	−0.08*π*
**DP**	320°	333°	0°	34°

## Discussion

### LGN activity

In our biological plausible model, competition and cooperation principles help in growth and decay of synaptic strengths. Both competition (reaction) and cooperation (diffusion) involves release of neurotrophic factors which are activity-dependent [55]–[58]. So our model requires neural activity.

During development, neural activities within both ON- and OFF-center pathways [92] are required for development of orientation selectivity. Retinal waves [93] appear too early and are unlikely to be directly responsible for establishing orientation selectivity [94], [95]. Taken together that eye specific segregation and ON/OFF segregation in LGN has already occurred [95] before the development of orientation selectivity and pharmacological blockade of ON center activity during development prevents maturation of orientation selectivity [94], suggest that LGN activity plays a role in development of orientation selectivity.

Weliky & Katz's [59] multi-electrode recording from anaesthetized ferret pups (P24-P27) prior to eye opening reveals correlated neuronal firing among LGN cells. The correlated neuron firing possess: (i) High correlation between same centre-type (ON-ON or OFF-OFF) neurons in same eye specific layer, (ii) Weak correlation between opposite centre-type (ON-OFF or OFF-ON) neurons in same eye-specific layer, and (iii) Weak but still significant correlation between left eye and right eye specific LGN layers. High correlation between same center-type LGN cells and anti-correlation between opposite center-type LGN cells in the same eye specific layer is essential for ON/OFF subregion formation in RFs. In our model correlation between same center-type LGN cells and anticorrelation between opposite centre-type LGN cells are modeled through the diffusion term in LGN. Erwin & Miller [36], [39] in their model have also used high correlation between same center-type LGN cells and anticorrelation between opposite center-type LGN cells using correlation functions. However, the high spatial correlation between left and right eye specific layers used in their model resulted in identical left and right RFs with 0° phase shift between the RFs. For high spatial anticorrelation between left and right eye specific layers used in their model resulted in left and right RFs with 180° phase shift between them. Experimental evidence does not support such high spatial anticorrelation used. Erwin & Miller's [36], [39] model is an extension of Miller's single eye model [71]. Piepenbrock et al. [37], [38] had independently extended Miller's Model [71] for getting binocular RFs. They obtained exactly the same results as Erwin & Miller [36], [39] with left and right RFs having either 0° or 180° phase shift. These models do not address disparity selectivity.

Weak but still significant correlation between left eye and right eye specific LGN layers as observed by Weliky & Katz [59] determines the relation between left and right RFs. In our model we have captured such correlation between an LGN cell in left eye and the corresponding LGN cell in right eye specific LGN layer through 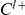 in equation (1). Our model yields disparity selective cells with phase shifts ranging from 0° to 180° between left and right RFs as reported in Ohzawa et al. [16].

We have used the following LGN cell activities during the RF development: (i) While updating a synaptic weight between a cortical cell and an LGN cell that particular LGN cell must be active. For instance while updating synaptic weight from the ON center LGN cell at position ‘J’ in left eye specific LGN, we put that LGN cell activity 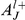 = 1. (ii) Activity of the LGN cell (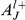) during synaptic weight update is determined by LGN spontaneous activity pattern as modeled by Goodhill [72]. If an LGN cell is inactive during weight update then the corresponding synaptic weight may decay unless helped by neighboring same-type cells.

**Figure 10:**
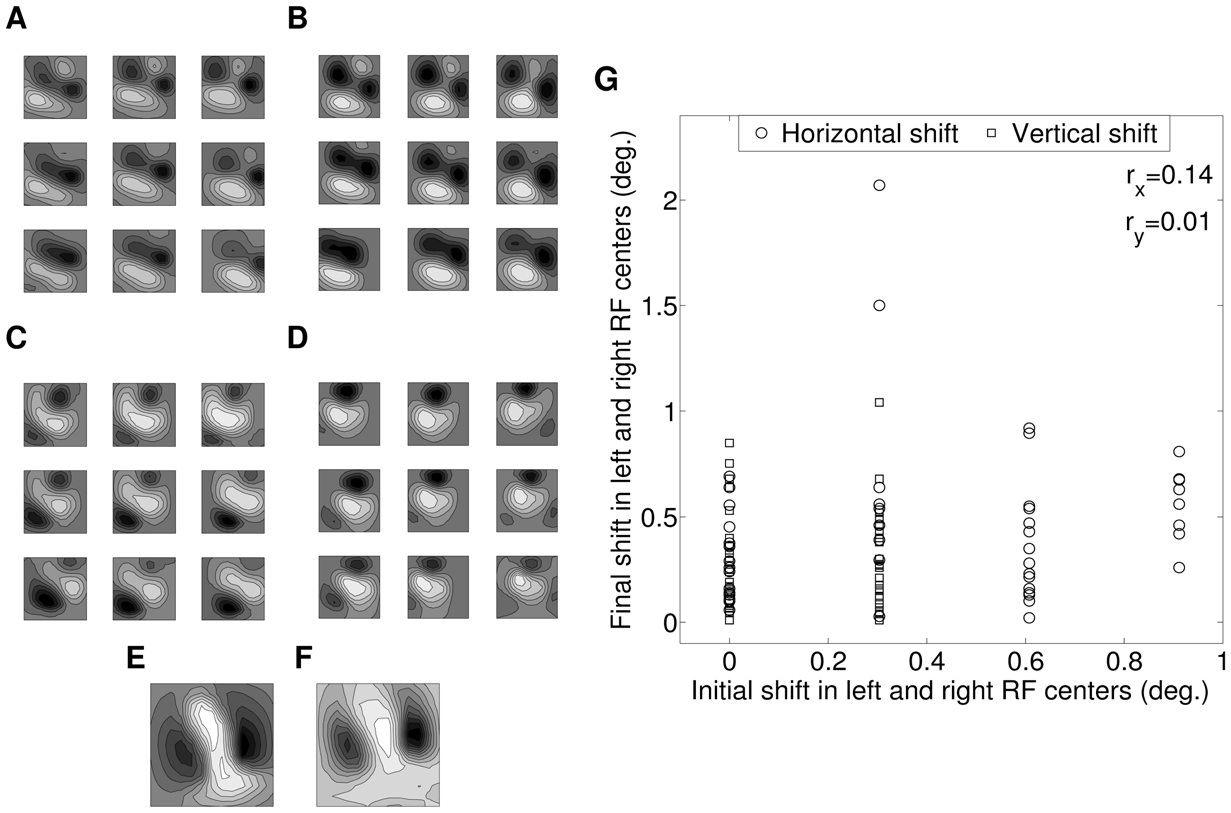
LGN cell activity, RF from Reverse correlation and Effect of initial RF center distribution. (A, B) 3×3 section of 2D left and right RFs from our model cortex. RFs are developed using the hypothesis that LGN cells are active during weight update. (C, D) 3×3 section of 2D left and right RFs from our model cortex. RFs are developed using LGN cell spontaneous activity as modeled by Goodhill [72]. (E) RF of a cortical cell as the set of LGN weights. (F) RF obtained using reverse correlation for the same cortical cell chosen in ‘E’. The RFs in ‘E’ and ‘F’ looks qualitatively similar. (G) Scatter plot of final versus initial shifts (horizontal and vertical) in VA of left and right RF centers for 50 cortical cells from our model cortex.

Figure 10A and B depict 3×3 sections of left and right RFs from 50×50 model cortex. These RFs are developed using the hypothesis that during synaptic weight update the concerned LGN cell is always active. We have also developed RFs using LGN cell spontaneous activity as modeled by Goodhill [72]. Figure 10C and D depict such left and right RFs. Diffusion in LGN, helps neighboring synapses within a cell RF to be of same type forming ON/OFF subregion. Cortical diffusion helps neighboring cortical cells to have similar response properties as seen in Figure 10. The nature of RFs developed remains same qualitatively for both types of LGN cell activity. This shows the robustness of our model for RF development.

To achieve computational speed, we employ LGN cell activity where we assume LGN cell to be active during weight update for the results presented in this article.

### RF from reverse correlation

Classically the RF is defined as a region of space where a visual stimulus can evoke a change in the firing activity of the cell. Experimentally RFs are mapped using reverse correlation technique, where the stimuli shown are correlated to the spikes obtained from the cell. Throughout this article we have represented left and right RFs as a set of LGN weights to a cortical cell. Now, we ascertain the validity of such representation by comparing RFs obtained by employing reverse correlation technique [96] (see File S1) with RFs obtained as a set of LGN weights. Figure 10E shows the RF of a cortical cell as the set of LGN weights. Figure 10F shows the RF obtained using reverse correlation for the same cortical cell chosen in Figure 10E. The RFs in Figure 10E and 10F look qualitatively similar. We therefore represent RFs as a set of LGN weights to a cortical cell throughout this article and save computational time to map RF using reverse correlation technique.

### Effect of initial RF center distribution

At the start of our simulation for RF development, left and right RFs centers were randomly shifted relative to each other with horizontal (H) shifts (-3≤H≤+3) and vertical (V) shifts (-1≤V≤+1) satisfying H∶V ratio of 3∶1 as reported in cat [7]. The initial assignment of horizontal and vertical shifts between left and right RF center positions does not significantly contribute to the difference in locations of left and right RF center in our developed cortical cells. To substantiate this, we fit 2D Gabor function to left and right RF profiles of developed cortical cells and obtain RF centers. Figure 10G shows a scatter plot of final and initial shifts in left and right RF centers for 50 simulated cortical cells. The correlation between final and initial horizontal shifts in left and right RF centers is r_x_ =  0.14. The correlation between final and initial vertical shifts in left and right RF centers is *r_y_* =  0.01. The weak correlation indicates that the final shifts in left and right RF centers do not depend on initial assignments.

### IDPO

Left and right retinal images can be well described in terms of positional and phase disparities. This does not necessarily mean that binocular disparity is encoded only through these cues. The visual system may employ any other feature as cue for depth, which represents a difference between left and right retinal images. For instance, small orientation difference between left and right retinal images may act as a cue for depth perception. Humans perceive depth when two lines of different orientations are presented in their left and right eye [97]. Cortical cells possess difference in their left and right eye preferred ORs. This neuronal property is referred as interocular difference in preferred ORs (IDPOs) [2] and can act as a cue for depth perception. Blakemore et al. [4] have reported a range of ±15° (S = 6–9°) IDPOs in cat. Bridge & Cumming [2] have reported a range of ±20° (S = 9.22°) IDPOs in macaque. In our model cortex, 69.3% OR tuned cells (1200 out of total 1732) have IDPOs range of ±20° (S = 9°). Rest 30.7% of cells have significant IDPOs (>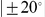).

### Orientation anisotropy

RF phase disparity shows orientation anisotropy. Cortical cells with vertical OR preference shows a wider range of RF phase disparity as compared to cells with horizontal OR preference. Figure 8A in Anzai et al. [1] depicts 97 disparity selective cells. Out of these 97 cells 16 cells have near-horizontal OR preference (0°±10°) and 11 cells have near-vertical OR preference (90°±10°). The cells with near-horizontal OR preference have phase disparity in the range of 0°–90° PA as compared to 0°–135° PA phase disparity range in the cells with near-vertical OR preference. In our model cortex, we have obtained RF positional and phase disparities for 415 cells. Out of these 415 cells, 36 cells have near-horizontal OR preference (0°±10°) and 55 cells have near-vertical OR preference (90°±10°). Majority (27 out of 36) of cells with near-horizontal OR preference cells have phase disparity in the range of 0°–45° PA and the rest of the cells have phase disparity range of 45°–90° PA. For near-vertical OR preference cells, phase disparity lies in the range of 0°–135° PA. Orientation anisotropy of phase disparity in our simulated cells is compared with Anzai et al.'s [1] data in Table 4.

**Table 4:** OR anisotropy.

Cell population data results			
		No. of cells (%)	
OR preference	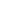 Range of RF phase disparity 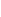	Anzai et al. [1] *	Our model**
near-vertical (90°±10°)	0°−45°	5 (45.4)	40 (72.73)
	45°−90°	3 (27.3)	11 (20)
	90°−135°	3 (27.3)	4 (7.27)
near-horizontal (0°±10°)	0°−45°	11 (68.75)	27 (75)
	45°−90°	5 (31.25)	9 (25)

*Total no. of cells = 97.

**Total no. of cells = 415.

### Disparity and Ocular Dominance

A cortical neuron acting as disparity detector, in principle should receive thalamic inputs from both the eyes. This principle was further verified by misaligning the two eyes during the postnatal critical development period. The misalignment causes cortical neurons to lose their OD and become completely ocular exclusive (monocular), leading to stereo blindness [98]. Single-unit electrophysiological studies using bar visual stimuli explored whether OD predicts disparity selectivity or sensitivity at single cell level [8], [11], [83], [99]. These studies show conflicting results. Thus no consensus could be reached on the OD and disparity selectivity relationship. Single-unit electrophysiological studies using random-dot stereogram (RDS) visual stimuli in awake macaque V1 reports that OD and disparity selectivity are not related to one another [28], [29]. Recent two-photon calcium imaging studies in area 18 of anaesthetized cat using drifting sinusoidal grating visual stimuli also obtained no relationship between OD and disparity selectivity or sensitivity [24]. In our model cortex, we ascertain the relationship between OD and disparity sensitivity (or selectivity) at cell population level and found that they are unrelated to one another (r = 0.02) as depicted in the Figure 4D.

### Significance

This paper, to the best of our knowledge, first time present a model on the development of receptive field for disparity selective simple cells and development of disparity map. We model disparity selectivity in layer IV of cat V1 using reaction-diffusion two-eye paradigm. In this model the wiring between LGN and cortical layer IV is determined by resource an LGN cell has for supporting connections to cortical cells and competition for target space in layer IV.

In our modeled cortex 48.6% cells show disparity selectivity for vertical surfaces, 49.5% cells show dif-frequency selectivity i.e. these cells encode depth for slanted surface and 30.7% cells show significant IDPOs. Disparity selective cells for vertical surfaces have RF properties such as (i) matched OR preference within ±15° interocular difference [2], [4], (ii) ocularly matched SF preference within ±0.05° cycles/degree [18], [19].

At map level, our model yields disparity map in conjunction with OR and OD maps. OD peak points lie on/near the pinwheel singularities of OR map. The disparity map is weakly clustered together like the reported data on monkey [28]. The disparity map can be used to study how complex cells in V1 pool activities of multiple simple cells. How and to what extent complex cells pool activities of simple cells is fundamental to the understanding of how progressively more complex selectivity in higher visual cortical areas develops.

The results presented in this article pertain to pre-eye opening development of disparity selectivity. Our model can be used for post-eye development by assigning input activity 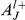 in equation (1) to represent natural images.

The model uses competition-cooperation based paradigm for development of wiring between two layers- in this case LGN and layer IV in V1. The model can be used with appropriate layer characterizations for studying development of connections in other areas of cortex.

## Supporting Information

File S1 **Details regarding Cortical cell response, RF mapping and Simple cell Characterization.**
(PDF)
