# Supplementary material for: A Reaction-Diffusion Model to Capture Disparity Selectivity in Primary Visual Cortex
Source: PLoS One. 2011 Oct 13;6(10):e24997. doi: 10.1371/journal.pone.0024997 (PMC3192717; doi:10.1371/journal.pone.0024997)
Supplement: File S1 — Details regarding Cortical cell response, RF mapping and Simple cell Characterization. (PDF) [file pone.0024997.s001.pdf]

Spike response model (SRM) for cortical cell response

Membrane potential of the cortical cell is evaluated using that equation:

$$u_I(t) = \eta(t - t_{fC}) + \beta_I \left(\sum_J (W_{IJ}^l) \sum_{fL} \varepsilon^l (t - t_{fL}) + \sum_J (W_{IJ}^r) \sum_{fR} \varepsilon^r (t - t_{fR}) \right) + R_P$$

Supp Equation 1

where $u_I(t)$ is the membrane potential of cortical cell at location 'I' at time t. t_{fC} is the time when the cortical cell produced the most recent spike. W_{IJ}^l is the strength of the synapses between left ON/OFF LGN cell at location 'J' and cortical cell at location 'I'. W_{IJ}^r is the strength of the synapse between right ON/OFF LGN cell at location 'J' and cortical cell at location 'I'. $\varepsilon^l(t - t_{fL})$ is the EPSP (excitatory post synaptic potential) generated in the cortical cell at location 'I' when the left LGN cell at location 'J' produces spike at time t_{fL}. $\varepsilon^r(t - t_{fR})$ is the EPSP (excitatory post synaptic potential) generated in the cortical cell at location 'I' when the right LGN cell at location 'J' produces spike at time t_{fR}. We have summed up the effect of all the spikes of the left and right LGN cells occurring within the past 90 msec from the present time t. $\eta(t - t_{fC})$ is the refractory voltage of the cortical cell. For more details on ε^l, ε^r and η functions refer to Gerstner (1999). R_P is the resting potential of the cortical cell. For details regarding calculation of synaptic scaling factor β_I refer Bhaumik & Mathur (2003).

RFs mapped using reverse correlation

We use reverse correlation technique as employed in experimental studies to get a better picture of real visual RFs obtained in our model. So the modeled RFs were mapped using the reverse correlation technique (Jones and Palmer 1987). The visual area representing RF of the cell is divided into a 20 x 20 grid. Small bright and dark rectangular bar stimuli of size 3 x 1 grid units are shown in random order. The response of the cortical neuron as train of action potentials is noted. We choose a correlation delay of zero as our model does not have any delay in the feed forward path, from retina to cortex. And each spike is assigned to the stimulus that preceded it by correlation delay. Then for each stimulus that evoked a spike (response), a 2D histogram grid is incremented at the coordinates corresponding to each stimulus location by the number of times it generated a spike. We obtained two separate histograms for bright and dark bar stimuli's. Final RF is given by the difference of bright and dark histograms.

Developed simple cells characterization and maps

Determination of OR selectivity, OD and SF

Left monocular responses of cortical cells are obtained by stimulating the left retina with sinusoidal gratings of different ORs varying from 0° to 180° in steps of 18°, and right retina with zero input. Similarly, right monocular response is obtained by stimulating the right retina. Binocular responses of cortical cells are obtained by stimulating both left and right retinae with sinusoidal gratings of different ORs varying from 0° to 180° in steps of 18°. A third order spline curve is fitted through the ten responses of cortical cell (called as OR tuning curve) to obtained half width at half height (hwhh) of a cortical cell. Thus, we obtained binocular, left monocular and right monocular OR selectivity and hwhh for all cortical cells of our 50 x 50 cortex. With this we can plot left, right and binocular OR maps.

OD is computed using the expression given by Albus (1975). OD=(R_r - R_l)/(R_r + R_l), where R_r (R_l)is the sum of the right (left) monocular responses of cortical cell to sinusoidal grating input of different OR. OD map is obtained by plotting OD across the 50 x 50 cortex. We measure SF tuning of each cortical cell by stimulating our modeled retina by sinusoidal grating having spatial frequencies varied from 0.1 to 1 cycle per degree in steps of 0.1 with OR of the grating fixed at the cell's preferred OR. Then a third order spline curve is fitted to these responses (called spatial frequency tuning curve) to obtained optimal SF. The optimal SF is the spatial frequency at which the cell responds most vigorously.

Determination of RF position and phase disparities

We fit 1D Gabor function to 1D RF profile (see Results section for obtaining 1D RF profiles) of left and right RFs to compute RF position and phase disparities. Gabor function fitted to 1D RF profile of left RF has the form:

$$G^l(x) = A_m \exp\left(\frac{-(x-x_o^l)^2}{(\sigma/2)^2}\right)\cos\left(2\pi f_l\left((x-x_o^l)+\frac{\phi_l}{2\pi f_l}\right)\right)+A_o$$

Supp Equation 2

where 'x' is RF size width in visual angle (VA). We have used fixed RF size of approximately 4° (width) x 4° (height) visual space. Gabor function has six free parameters: (i) 'A_m', amplitude (ii) 'x_o^l', centre position of Gaussian envelope in VA (iii) 'σ', width of Gaussian (iv) 'f_l', spatial frequency of sinusoid in cycles per degree in VA (v) 'ϕ_l', phase of sinusoid in phase angle (PA) having range of -180° to 180° and (vi) 'A_o', amplitude offset. The offset amplitude is very close to zero and can be omitted from the parameter list. Nevertheless, it is considered in

fitting procedure because the data to be fitted always exhibit a non-zero value to this parameter. A similar Gabor function is fitted to 1D profile of right RF to obtain 'x_o^r', 'f_r', and 'ϕ_r'. Fitting is done by employing Levenberg-Marquardt fitting procedure. RF position disparity (δx_o) is defined as ($x_o^l - x_o^r$) in VA. RF phase disparity ($\delta\phi$) is defined as ($\phi_l - \phi_r$) in PA. RF phase disparity ($\delta\phi$) can also be expressed in VA as ($\frac{\phi_l}{360 f_l} - \frac{\phi_r}{360 f_r}$). We consider right eye as a reference to left eye for determining position and phase disparities (Tsao & Conway 2003). We define overall RF spatial disparity as $((-2\pi f_l x_o^l + \phi_l) - (-2\pi f_r x_o^r + \phi_r))$ in PA. This simplifies to $(\delta\phi - 2\pi f \delta x_o)$, where $f = f_l = f_r$.

Determination of preferred binocular phase disparity and disparity sensitivity

To determine preferred binocular phase disparity and disparity sensitivity of a cortical cell, modeled retinae are dichoptically stimulated with drifting sinusoidal gratings at cell's preferred OR and SF. The relative phase difference between the dichoptically shown sinusoidal gratings were varied from 0° to 360° in steps of 18° (total number (n_p) of relative disparity phases=20). The cortical cell responses at various relative disparity phases (n_p=20) are obtained and fitted with a cycle of sine wave using least square criterion to obtain a disparity tuning curve. The relative disparity phase at which the fitted sine wave peaks gives the preferred binocular phase disparity (DP). The ratio of the amplitude of a sine-fitted disparity tuning curve to its mean response amplitude is defined as disparity sensitivity or selectivity (DSen) (Kara & Boyd 2009) or Binocular interaction index (BII) (Ohzawa & Freeman 1986, Smith et al. 1997). A signal-to-noise ratio (S/N) is determined as the ratio of amplitude of fitted sine wave to the residual mean square error of the fit (Ohzawa & Freeman 1986, Smith et al. 1997). Cortical cells are classified as phase specific i.e. disparity selective if DSen$\geq$0.3 and S/N>2 else they are classified as non-phase specific (Ohzawa & Freeman 1986). Preferred binocular phase disparity and DSen can also be determined as follows by Kara & Boyd (2009) method. The cortical cell responses at various relative disparity phases (n_p=20) are converted into vectors ($\vec{V_i}$, i=1 to n_p) with magnitude equal to response amplitude and angle equal to relative disparity phase. Then the average resultant vector ($\vec{V}_{avg}$) is computed as $\vec{V}_{avg} = \sum_{i=1}^{n_p} \vec{V}_i / n_p$. Average response amplitude (A_{avg}) to various relative disparity phase (n_p=20) stimulation is given by $A_{avg} = \sum_{i=1}^{n_p} |\vec{V}_i| / n_p$. The angle of $\vec{V}_{avg}$ is the preferred binocular phase disparity. Disparity sensitivity (DSen) is calculated as $DSen = 2 |\vec{V}_{avg}| / A_{avg}$.

Albus K (1975) Predominance of monocularly driven cells in the projection area of the central visual field in cats striate cortex. Brain Research 89: 341-347.

Bhaumik B, Mathur M (2003) A cooperation and competition based simple cell receptive field model and study of feed-forward linear and nonlinear contributions to orientation selectivity. J Comp Neuroscie 14: 211-227.

Gerstner W (1999) Spiking neurons. In: Mass W, Bishop CM, editors, Pulsed Neural Networks. pp. 3-54.

Jones JP, Palmer LA (1987) The two-dimensional spatial structure of simple receptive fields in cat striate cortex. J Neurophysiol 58: 1187-1211.

Kara P, Boyd JD (2009) A micro-architecture for binocular disparity and ocular dominance in visual cortex. Nature 458: 627-632.

Ohzawa I, Freeman RD (1986) The binocular organization of simple cells in the cats visual cortex. J Neurophysiol 56: 221-242.

Smith EL, Chino YM, Ni J, Ridder WH, Crawford MLJ (1997) Binocular spatial phase tuning characteristics of neurons in the macaque striate cortex. J Neurophysiol 78: 351-365.

Tsao DY, Conway BR (2003) Receptive fields of disparity-tuned simple cells in macaque V1. Neuron 38: 103-114.
